# High-Cycle Fatigue Behavior and Corresponding Microscale Deformation Mechanisms of Metastable Ti55511 Alloy with A Basket-Weave Microstructure

**DOI:** 10.3390/ma15207144

**Published:** 2022-10-13

**Authors:** Hengjun Luo, Wuhua Yuan, Wei Xiang, Hao Deng, Hui Yin, Longqing Chen, Sheng Cao

**Affiliations:** 1 College of Materials Science and Engineering, Hunan University, Changsha 410082, China; 2 Deyang Wanhang Die Forging Co., Ltd., China National Erzhong Group Co., Deyang 618013, China; 3 Key Laboratory of Radiation Physics and Technology of Ministry of Education, Institute of Nuclear Science and Technology, Sichuan University, Chengdu 610064, China; 4 Department of Mechanical Engineering, College of Engineering, Shantou University, Shantou 515063, China

**Keywords:** Ti-5Al-5Mo-5V-1Cr-1Fe alloy, high-cycle fatigue, basket-weave microstructure, deformation mechanisms

## Abstract

High-cycle fatigue (HCF) is a critical property of metastable β Ti alloys in aerospace applications. In this work, the HCF behavior and corresponding microscale deformation mechanisms of a metastable Ti-5Al-5Mo-5V-1Cr-1Fe (Ti55511) alloy with a basket-weave structure were investigated. HCF and its deformation mechanisms of a Ti55511 alloy were systematically studied in the deformed condition by using a scanning electron microscope (SEM), a transmission electron microscope (TEM), and electron backscatter diffraction (EBSD). It was found that the Ti55511 alloy exhibited an excellent HCF strength (10^7^ cycles, K_t_ = 1, R = 0.06) of 738 MPa. The fractographic investigation demonstrated that fatigue striations and secondary cracks were the main features in the crack initiation zone. Dislocation analyses indicated that the HCF deformation of the basket-weave microstructure was mainly affected by the dislocation slipping of the primary α (α_p_) phase. In addition, the dislocation pile-up at the α_p_/β_trans_ interface led to crack initiation. EBSD analyses indicated that the prismatic *<a>* type slip system of the α_p_ phase was preferentially activated during the HCF deformation process of the Ti55511 alloy, followed by the basal *<a>* type and pyramid *<a>* type systems.

## 1. Introduction

The Ti-5Al-5Mo-5V-1Cr-1Fe (Ti55511) alloy is a typical metastable β titanium alloy with high strength and excellent fracture toughness [1,2]. Therefore, Ti55511 is widely used in aviation structural components, such as landing gear [3]. It is well accepted that a variety of microstructures of Ti alloys can be obtained according to the specific design of the thermomechanical processing and heat treatment strategies, such as a bimodal microstructure, a basket-weave microstructure, and a Widmanstätten microstructure [4]. These microstructures possess different features, such as the morphology of the α phase, which further cause varied mechanical properties. Among these microstructures, the basket-weave microstructure features the combination of a lamellar primary α (α_p_) and fine secondary α (α_s_). In addition, the basket-weave microstructure is the most frequently used microstructure in titanium alloys due to its excellent fracture toughness and crack propagation resistance [5,6]. Corresponding investigations are necessary especially in the aviation and aerospace industries.

As for aviation applications, high-cycle fatigue (HCF) is a critical property of structural components made with titanium alloys with a basket-weave microstructure [4], considering that most of the structural components are subjected to cyclic loading during service and are prone to fail [7]. Many studies have been conducted on the HCF behavior of Ti55511 alloys. Shi and coworkers investigated the initiation of HCF cracks in a Ti55511 alloy with a basket-weave microstructure, showing that the presence of the coarse α prompted the crack initiation [8]. Wu’s group proposed that the area of the crack initiation region in Ti55511 decreased in the order of coarse basket-weave microstructure, fine basket-weave microstructure, Widmanstätten microstructure, and bimodal microstructure [9]. The structural types with a high fatigue strength corresponded to a small initiation area. The fracture behavior of a Ti55511 alloy with a basket-weave microstructure was studied by Liu et al. based on various testing parameters (e.g., stress concentration factors K_t_ and stress ratios R) [10]. The results showed the HCF strength of the Ti55511 alloy increased with the increase in R and decreased with the increase in K_t_. Other studies also reported the HCF properties of Ti55511 variant alloys (e.g., Ti-5Al-5Mo-5V-3Cr and Ti-5Al-5Mo-5V-3Cr-1Zr) with different microstructures. Huang et al. explored the HCF properties of the Ti55531 alloy with bimodal and lamellar microstructures and revealed the crack initiation behaviors and α phase deformation mechanism [11,12]. The effect of the continuity of the grain boundary α (GBα) on the HCF properties of the Ti5553 alloy after STA and BASCA heat treatments was clarified by Bolfarini and coworkers [13]. The microstructural characteristics (especially the morphology and size of α phase) significantly influenced the HCF property of Ti55511 or similar metastable Ti alloys, since the deformation during HCF was mainly caused by the dislocation slip of the α phase. The α phase of titanium alloy is a closed packed hexagonal crystal structure containing both *<a>* and *<c+a>* slip systems, which can be activated depending on the deformation condition. During the HCF process at microscale, however, relevant studies have rarely been performed and are urgently required to determine the deformation mechanisms in the Ti55511 alloy.

Herein, the deformation mechanism of the Ti55511 alloy with a basket-weave structure under HCF loading was investigated. In detail, the deformation behaviors of the α_p_ and α_s_ phases in the Ti55511 alloy under HCF loading were systematically studied using SEM, TEM, and EBSD techniques. This work is expected to provide theoretical and experimental support for understanding the dislocation slip mechanism of the basket-weave structure in β-type titanium alloys during HCF deformation.

## 2. Materials and Methods

**Figure 1:**
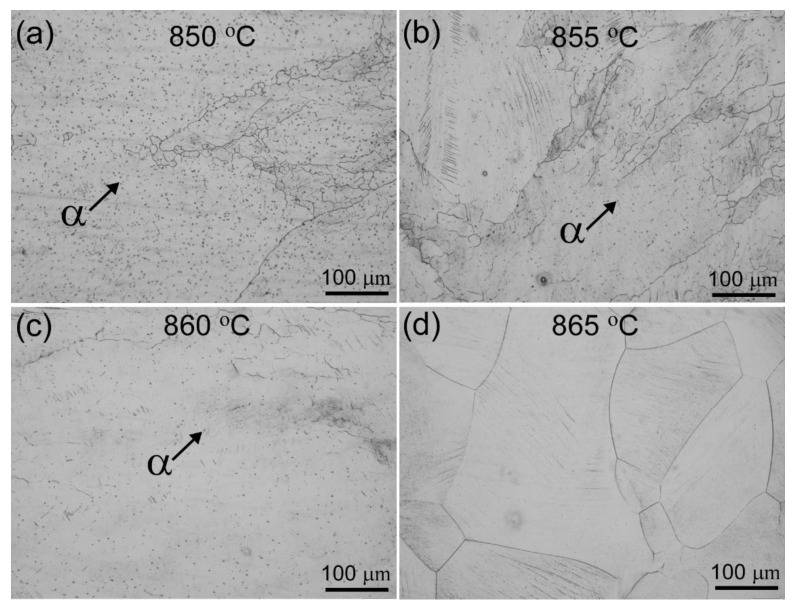
Optical micrographs of the water-quenched Ti55511 alloy at different temperatures: (**a**) 850 ℃, (**b**) 855 ℃, (**c**) 860 ℃, and (**d**) 865 ℃.

**Figure 2:**
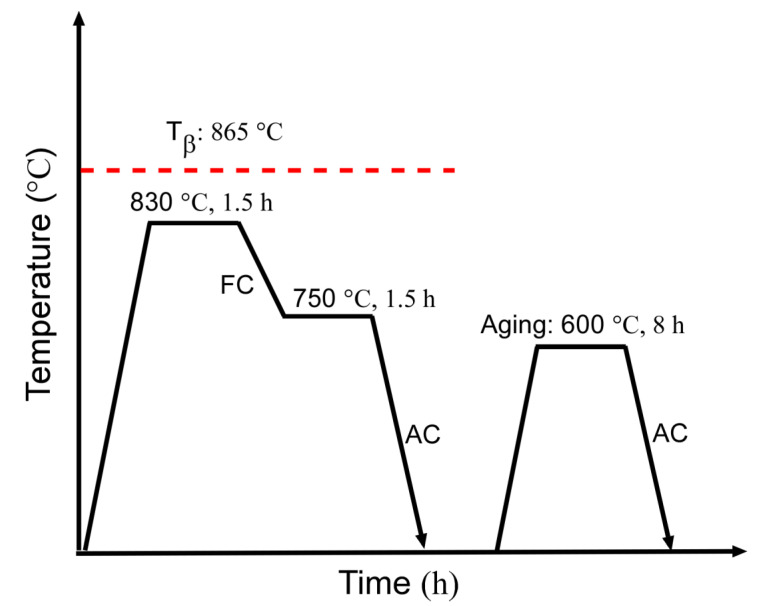
Schematic of the STA heat treatment.

The Ti55511 alloy was produced by Hunan Xiangtou Goldsky New Materials Co., Ltd. The chemical composition of the Ti55511 alloy was determined to be Ti-5.10Al-4.98Mo-4.95V-1.02Cr-0.98Fe-0.15O-0.005N (wt%). The β transus temperature (T_β_) was about 865 °C, which was measured by the metallographic method. The metallographic method determining the β transus included microscopic observation of the phases present in heat-treated and quenched samples from temperatures below and above β transus. As shown in Figure 1, the α phase was detectable below 865 °C and absent once 865 °C was reached. To obtain the basket-weave microstructure, Ti55511 was forged at 900 °C. Subsequently, the as-forged Ti55511 alloy was subjected to a solution-treated and aging (STA) heat treatment as shown in Figure 2. In detail, the as-forged Ti55511 alloy was firstly heat treated at 830 °C for 1.5 h, then furnace cooled (FC) to 750 °C for 1.5 h, and finally air cooled (AC) to room temperature. The aging process was performed at 600 °C for 8 h.

Microstructure characterizations were carried out using optical microscopy (OM, DMi8 M/C/A, Leica, Frankfurt, Germany), SEM (Inspect F50, Phenom-World, Eindhoven, Netherlands) with EBSD (NordlysNano, Oxford instrument, Oxford, UK), and TEM (F20, FEI, Phenom-World, Eindhoven, The Netherlands). The OM and SEM samples were ground and polished by using SiC paper to 3000 mesh and then etched with Kroll’s agent (1 vol% HF + 4 vol% HNO_3_ + 95 vol% H_2_O). The TEM sample was prepared by ion milling (PIPS II 695, Gatan Inc., Pleasanton, CL, USA). The EBSD sample was polished using a vibration polisher (VibroMet 2, Buehler, Lake Bluff, IL, USA). The accelerating voltage, current, and step size during the EBSD test were set at 20 kV, 2.4 mA, and 2.0 μm, respectively. Channel 5 software (Version 7.2, Oxford instrument, Oxford, UK) was used for EBSD data processing.

**Figure 3:**
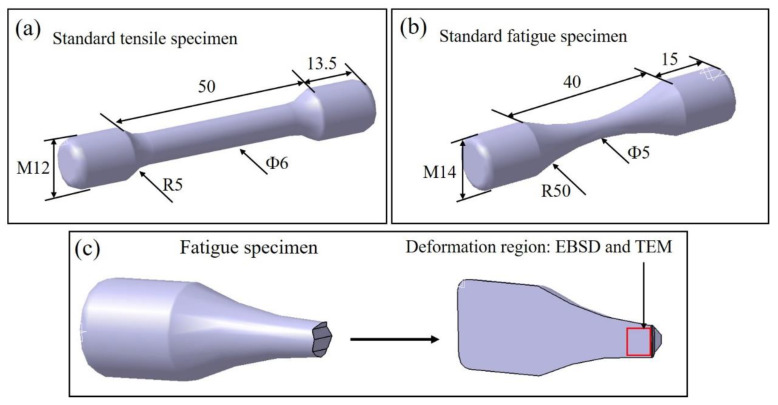
(**a**) The standard cylindrical tension test samples, (**b**) the standard cylindrical fatigue test samples, and (**c**) the sampling location for the EBSD and TEM tests of the fatigue sample.

Tensile tests were conducted on an Instron 8801 machine (Instron, Norwood, MA, USA) equipped with an extensometer. In addition, standard M10 cylindrical tension test samples were cut according to the ASTM E8/E8M-16a standard, as shown in Figure 3a. The tensile strain rate was 0.5 mm/min. Two tensile testing samples were tested to determine the average values of the ultimate tensile strength σ_u_, yield strength σ_y_ and elongation. HCF tests (at 10^7^ cycles) were performed on an MTS Landmark fatigue machine in air with R = 0.06, K_t_ = 1, and a frequency of 100 Hz, according to the ISO12107 standard. The size of the standard HCF samples is presented in Figure 3b. To clarify the actual deformation mechanism of the HCF specimen, an area of interest (AOI) was selected on the longitudinal cross section of a fresh fracture surface for EBSD and TEM characteristics (Figure 3c).

## 3. Results and Discussion

### 3.1. Microstructure of the Ti55511 Alloy

**Figure 4:**
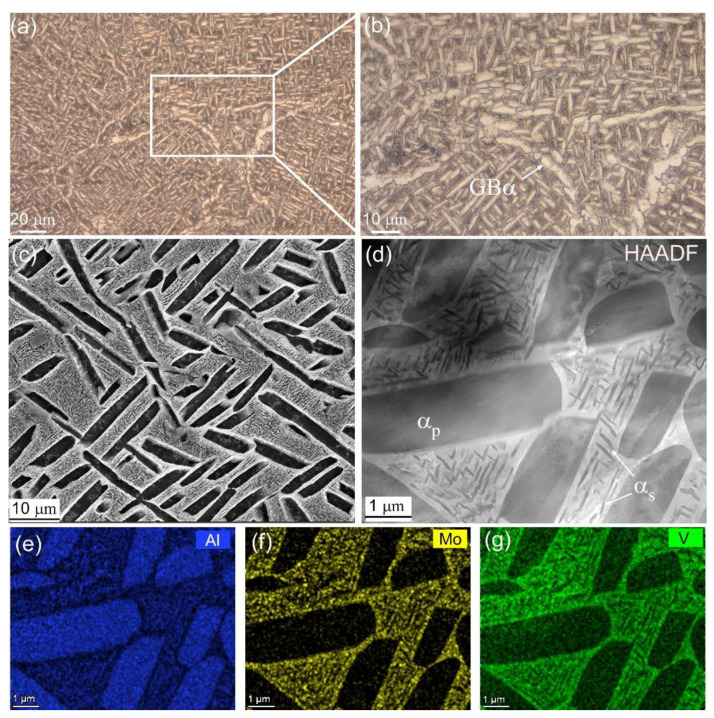
(**a**,**b**) OM images of the basket-weave microstructure, (**c**) SEM secondary electron image of the Ti55511 alloy with a basket-weave microstructure, (**d**) TEM-HADDF image showing the morphologies of the α_p_ phase and the β_trans_ structure, and (**e**–**g**) the STEM-EDS results collected in (**d**).

Figure 4 shows the microstructural details of the Ti55511 alloy. Based on the OM images (Figure 4a,b), the microstructure of the Ti55511 alloy after STA heat treatment was a typical basket-weave microstructure consisting of the GBα, α_p_ lamella, and β transformed microstructures (β_trans_). The GBα was discontinuous, which ensured the adequate ductility of the Ti55511 alloy. The SEM secondary electron image in Figure 4c clearly displays the rod-like morphology of the α_p_ phase. According to the statistical results, the average width of the α_p_ lamella was 1.08 μm. In addition, the β_trans_ microstructures were recognized among the α_p_ phases, composed of retained β and α_s_ phases. In Figure 4d, the TEM high-angle annular dark field (HAADF) image distinctly illustrates the existence of the α_s_ with the length ranging from 200 nm to 700 nm. The corresponding STEM-EDS results indicated that the Al (α stabilizer) mainly diffused into the α phase (Figure 4e), while the Mo and V (β stabilizers) permeated into the β phase (Figure 4f,g).

### 3.2. Tensile and HCF Properties of the Ti55511 Alloy

**Figure 5:**
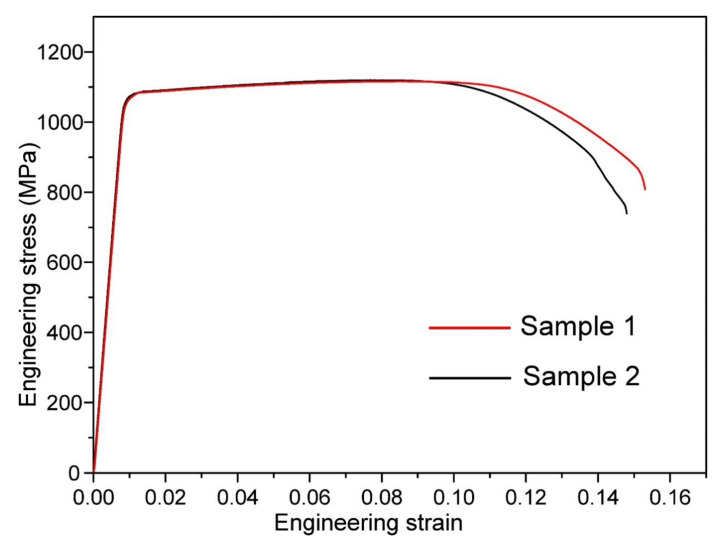
Engineering stress–strain curves of the Ti55511 alloy.

**Figure 6:**
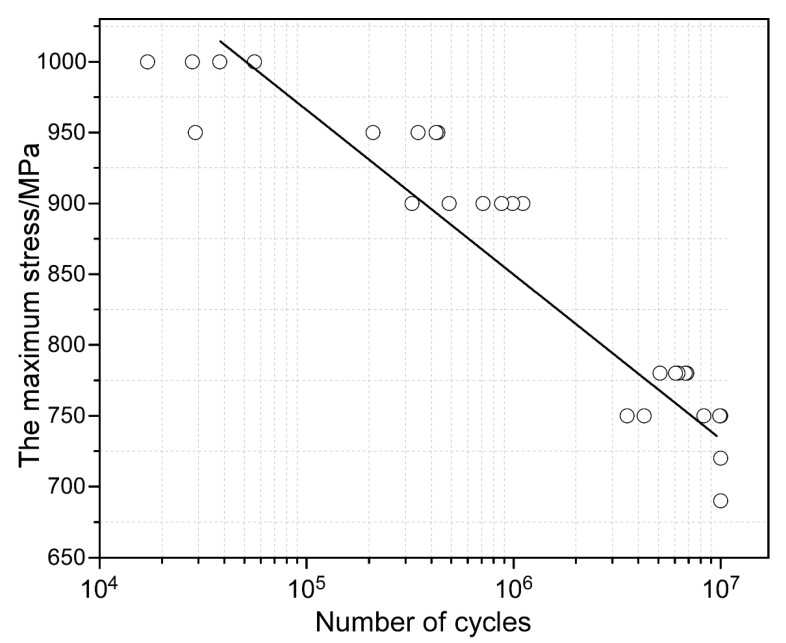
S-N curve of the Ti55511 alloy.

The engineering stress–strain curves of the Ti55511 alloys are provided in Figure 5. For better reliability, the same samples were tested twice to obtain an average value. In detail, the Ti55511 sample with the basket-weave microstructure exhibited an averaged σ_u_ of 1119 MPa, a σ_y_ of 1098 MPa, and an elongation of 13.8%. Figure 6 plots the S-N curve of the Ti55511 alloy according to the HCF test data, and the HCF limit (σ_max_) was fitted to be 738 MPa. Some researchers have proposed that the HCF limit is closely related to the yield strength [12,14,15]. As the HCF is mainly controlled by the crack initiation, the HCF property is significantly influenced by the dislocation glide and is generally related to the yield strength [14]. In general, alloys with high yield strength would have a high HCF strength. For example, the β CEZ alloy had a high HCF strength (575 MPa) and a high σ_y_ (1190 MPa), with a σ_max_ /σ_y_ ratio at 0.48. In contrast, the Ti-6Al-4V alloy had a σ_max_ /σ_y_ ratio at 0.42 (σ_max_: 375 MPa and σ_y_: 915 MPa) [14]. Previous studies suggested that the beta CEZ had a superior fatigue performance to the Ti-6Al-4V, as it had a higher σ_max_ /σ_y_ ratio [14]. Previous work has shown that the σ_max_/σ_y_ value of the metastable β Ti alloys (e.g., Ti55531, βCEZ, etc.) was 0.49~0.507 [12,14,15]. In our work, the value of σ_max_/σ_y_ reached 0.672, which proved that the Ti55511 alloy with a basket-weave microstructure had excellent HCF properties.

### 3.3. The Fracture Surface of the HCF Sample

**Figure 7:**
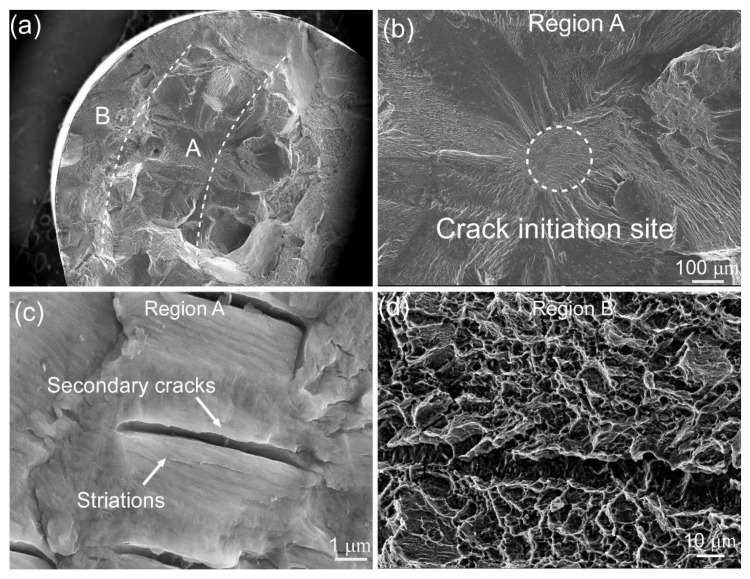
The longitudinal cross section and the fracture surface of the Ti55511 fatigue specimens (loading, 750 MPa; life, 8.29 × 10^6^ cycles). (**a**) Macroscopic fracture surfaces, (**b**) the crack initiation site in Region A, (**c**) fatigue striations and secondary cracks in Region A, and (**d**) the dimple structures in Region B.

In order to analyze the fracture behavior of the Ti55511 alloy during the HCF process, the longitudinal cross section and the fracture surface of the fatigue specimens (loading 750 MPa, life 8.29 × 10^6^ cycles) were characterized with SEM at different magnifications, as shown in Figure 6. The macroscopic fracture surfaces of the HCF specimen were observed, as shown in Figure 7a; the crack initiation sites (Region A) of the Ti55511 specimen can be recognized easily on the fracture surfaces. Moreover, the shear lip zone (Region B) spread around the crack initiation site. Figure 7b presents the enlarged image of the crack initiation sites marked in Figure 7a. The crack initiation site was located inside the HCF specimen, which was similar to previous studies on the other β Ti-alloys [11,16,17]. According to the results presented by Shi et. al. [8], during the HCF test of the Ti55511 alloy, the crack initiation site was usually located at the blocky α_p_ boundary (which was considered a weak point) when the crack source appeared inside the sample (rather than on the surface). In addition, many fatigue striations and secondary cracks were observed in Region A, as displayed in Figure 7c. The distance between the secondary cracks was similar to the width of the α_p_ phase. Therefore, it is proposed that the striations were mainly generated in the α_p_ lath. Furthermore, enormous dimples appeared in Region B (Figure 7d), which is a typical feature of a ductile fracture mode.

### 3.4. The Deformation Mechanism of the HCF Sample

**Figure 8:**
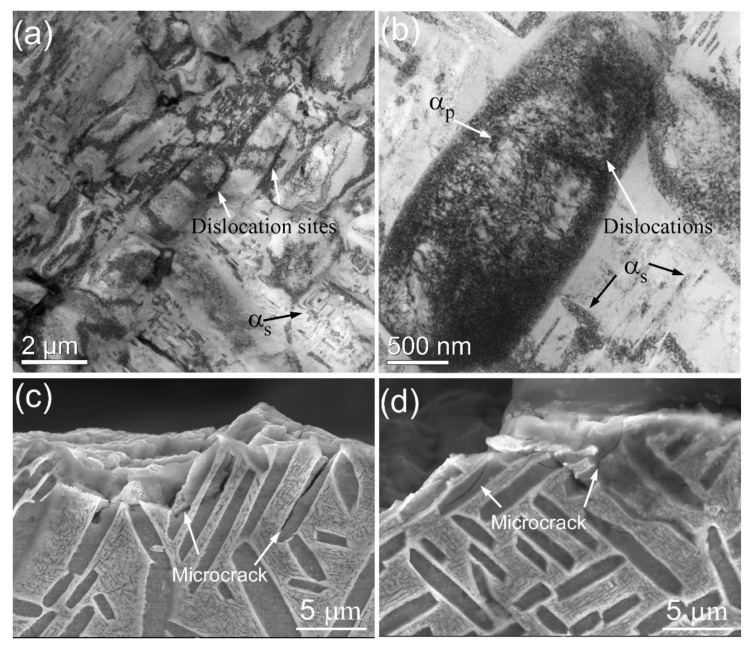
(**a**,**b**) TEM images showing the dislocations in the α_p_ and α_s_ phases; (**c**,**d**) cross-sectional fractographs of the Ti55511 fatigue specimen (loading, 750 MPa; life, 8.29 × 10^6^ cycles).

The TEM images of the Ti55511 fatigue specimen (loading 750 MPa, life 8.29 × 10^6^ cycles) are presented in Figure 8. Many dislocations were observed in the α_p_, according to Figure 8a,b. For the Ti alloys, α_p_ is a relatively soft phase, and cracks driven by fatigue loading would firstly activate at the α_p_/β_trans_ interface [18]. In general, the relatively large size of the α_p_ phase provided enough room for dislocation slipping. Therefore, massive dislocations were generated and entangled in the α_p_ phase. In addition, the dislocation structure was also recognized in the fine α_s_ phase, demonstrating that the α_s_ phase did bear part of the deformation. In order to analyze the crack initiation behavior, the cross section of the fatigue specimen (loading 750 MPa, life 8.29 × 10^6^ cycles) near the crack initiation sites was characterized by SEM, as shown in Figure 8c,d. The results showed that the microcracks were mainly initiated at the α_p_/β_trans_ interface during fatigue deformation. For the basket-weave structure, previous studies have observed crack initiation at the α_p_/β_trans_ interfaces [8]. It is attributed to the local dislocation pile-up, which will lead to stress concentration and the formation of microcracks.

**Figure 9:**
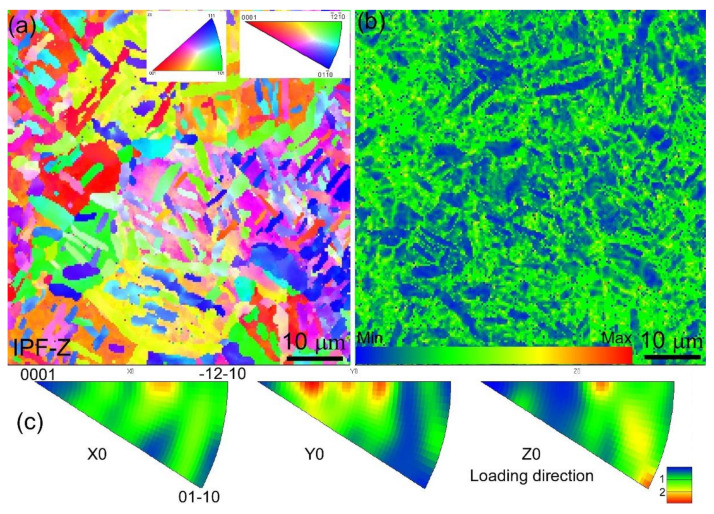
(**a**) EBSD orientation maps collected at AOI, (**b**) and (**c**) are the corresponding KAM map and IPF, respectively.

To investigate the microscale deformation mechanisms of the Ti55511 alloy under fatigue loading, EBSD was employed to analyze the fatigue specimen (loading, 750 MPa; life, 8.29 × 10^6^ cycles). Since the deformation of the Ti55511 alloy was mainly provided by the α_p_ phase under the HCF test process, we only focus on the deformation mechanism of the α_p_ phase in this work. The EBSD results of the AOI are shown in Figure 9. Figure 9a illustrates the orientation maps in the loading direction (Z0). The α_p_ phase was randomly distributed on the β matrix, which was consistent with the OM and SEM results. The EBSD kernel average misorientation (KAM) map is a powerful tool to probe local lattice distortion and qualitatively evaluate deformation uniformity. Figure 9b shows the EBSD-KAM map of the Ti55511 HCF specimen. The distinct intensity difference reflected the uneven deformation under the HCF load, which originated from the fact that the α_p_ phases with random orientations exhibited various degrees of deformation. Indeed, the inverse pole figures (IPF), shown in Figure 9c, also displayed the state of random orientation, confirming the absence of a preferred orientation in the AOI region.

**Figure 10:**
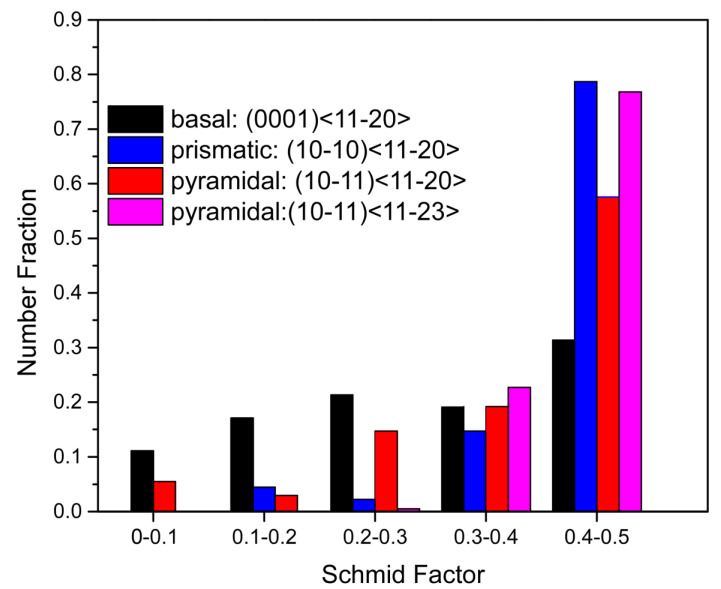
Statistics of the identified slip systems of the α_p_ phase at the AOI.

The various slip systems for the α titanium were *<a>* and *<c+a>* types. In detail, the *<a>* type slip systems included basal (0001) <11–20>, prismatic (10–10) <11–20>, and pyramidal (10–11) <11–20>, while the *<c+a>* type slip system contained (10–11) <11–23>. The activation of the slip systems can be determined by the Schmidt law, i.e., once the critical resolved shear stress (τ_crss_) of a specific slip system is exceeded by the resolved shear stress on the slip plane along the slip direction, the initiation of yielding in a crystal takes place [19]. According to the Schmidt law, the τ_crss_ can be calculated by the Equation (1) [20]:

(1) $$\tau_{crss}=\sigma_{y}.m$$

where σ_y_ is the yield strength of the material, and *m* is the Schmidt factor. In order to investigate the activation of the slip systems in the Ti55511 alloy, the Schmidt factor of different slip systems was calculated, as shown in Figure 10. Moreover, the τ_crss_ for different slip systems of the Ti alloys reported by previous studies are summarized in Table 1. For the prismatic *<a>* slip system, the statistical results showed that 78% of the α_p_ grains exhibited the values of the Schmidt factor at above 0.4. In comparison, the fraction of the α_p_ grains with a Schmidt factor at above 0.4 in the basel *<a>* and pyramidal *<a>* were 40% and 60%, respectively. More importantly, the τ_crss_ of the prismatic *<a>* slip system (~375 MPa) was slightly lower than that of the basel *<a>* slip system (~387 MPa) and was much lower than that of the pyramidal *<a>* slip system (~400 MPa), as listed in Table 1. Therefore, based on equation (1), it can be speculated that the prismatic *<a>* slip system was preferentially activated during the HCF deformation in most grains. The fraction of grains with basal and pyramidal *<a>* activation should be lower than prismatic *<a>*, as it had a slightly higher CRSS and lower volume fraction of grains with a Schmid factor above 0.4. Furthermore, the number of αp grains with a Schmidt factor >0.4 in the pyramidal *<c+a>* was identified as 77%, but the relatively high τ_crss_ (628 MPa) made it difficultto activate the pyramidal *<c+a>* slip system.

## 4. Conclusions

In this work, the basket-weave structure in the Ti55511 alloy was obtained by the STA heat treatment strategy, and the corresponding HCF properties were systematically studied. The main conclusions are as follows:

1. The basket-weave structure of the Ti55511 alloy consisted of discontinuous GBα, α_p_ lamellae, and a β_trans_ structure. The average width of the α_p_ was 1.08 μm, and the length of the α_s_ ranged from 200 to 700 nm.
2. The Ti55511 sample in this work exhibited an average ultimate tensile strength of 1119 MPa, a yield strength of 1098 MPa, an elongation of 13.8%, and an ultimate strength of HCF of 738 MPa.
3. The deformation mechanism of the Ti55511 during HCF was mainly determined by the dislocation slipping of the α_p_ phase, i.e., dislocations slip within the α_p_ phases. In addition, dislocation pile-up at the α_p_/β_trans_ interface induced stress concentration and microcrack initiation.
4. The EBSD analyses indicated the prismatic *<a>* type slip system of the α_p_ phase was preferentially activated during the HCF deformation process of the Ti55511 alloy, followed by the basal *<a>* and pyramid *<a>* systems, while the pyramid *<a+c>* slip was very difficult to activate.

## Figures and Tables

**Table 1:** The τ_crss_ for different slip systems of the α phase.

	**Basal*<a>***	**Prismatic*<a>***	**Pyramidal*<a>***	**Pyramidal*<c+a>***
CRSS	388 [21]	373 [21]	401 [22]	631 [22]
CRSS	373 [23]	355 [22]	395 [24]	612 [22]
CRSS	400 [25]	342 [23]	404 [24]	640 [25]
Mean value	387	375	400	628

## Data Availability

The data presented in this study are available on request from the corresponding author.
